# Chronic Pain and Residual Limb Outcomes Following Primary Major Lower Extremity Amputation vs. Minor-to-Major Amputation Progression: A Propensity Score-Matched Analysis

**DOI:** 10.3390/jcm15218393

**Published:** 2026-10-28

**Authors:** Matthew D. Ramsey, Jason Zhang, Sophia Bonfigli, Katherine Kozlowski, Lindsay E. Janes, Jason H. Ko

**Affiliations:** 1 Division of Plastic and Reconstructive Surgery, Department of Surgery, Northwestern University Feinberg School of Medicine, 259 E Erie St Ste 2060, Chicago, IL 60611, USA; matthew.ramsey@nm.org (M.D.R.); lindsay.janes1@nm.org (L.E.J.); 2 Department of Orthopaedic Surgery, Northwestern University Feinberg School of Medicine, Chicago, IL 60611, USA

**Keywords:** phantom, risk, revision, outcomes, mortality

## Abstract

**Introduction**: It is unclear if the pathway to major lower-extremity amputation (LEA) impacts development of post-amputation pain. This study compares pain outcomes between patients with minor LEA progressing to major amputation versus primary major LEA. **Methods**: Adults with major LEA (proximal to the ankle) were identified from the TriNetX Global Collaborative Network (2010–2026). Patients with an initial minor LEA (distal to the ankle) followed by a subsequent, distinct major LEA were compared with patients undergoing primary major LEA via propensity score matching across a total of 99 covariates. Patients with progression were further stratified using six intervals (0–3, 3–6, and 6–12 months, and ≥1, ≥2, and ≥5 years) corresponding to time between amputations. Outcomes were assessed using two-proportion z-tests and Kaplan–Meier time-to-event analysis. **Results**: Matched cohorts ranged from 997 to 6245 patients per group across the six intervals. Overall, patients with progressive minor-to-major LEA had highly significant, higher rates of residual limb pain (RLP) (risk difference range: 4.3–8.5%), phantom limb pain (PLP) (2.6–4.9%), and amputation revision or complications (10.9–17.2%) that persisted for five years after major amputation. Progression at 3–12 months displayed the highest risk differences for RLP (7.8–8.5%), progression within one year, the highest for revisions, while PLP displays an increased risk not dependent on progression interval. Progression was also associated with lower mortality if progression occurred more than 1 year from the minor amputation. **Conclusions**: Minor-to-major progression of LEA is associated with significantly worse chronic pain and amputation revisions in highly co-morbid patients. Progression represents a high-risk, and potentially high yield, population to target for earlier interventions to improve pain. RLP may be modifiable after the minor LEA to optimize outcomes after the major LEA while PLP appears to be driven primarily by the major LEA.

## 1. Introduction

Over 2 million individuals in the United States currently live with limb loss, with this number only expected to rise in the coming years. Lower-extremity amputations (LEAs) comprise over 90% of amputations performed, with the majority performed for diabetic and vascular complications [1,2]. Despite advancements in limb-salvage programs, the rates of LEA continue to rise, with a 50% increase from 2009 to 2015 in patients with diabetes [2]. An additional analysis of inpatient data projected an increase from 566,000 to 761,000 cases per year from 2019 to 2060 [1]. Furthermore, a study of lower-extremity amputations from 1993 to 2021 described increasing incidence of both below-knee amputations (BKAs) and above-knee amputations (AKAs), with a shift toward younger patients as the mean age for BKA decreased from 68 to 61.6 years and from 75 to 66.7 years for AKA [3].

These trends are clinically significant as many patients experience chronic pain after amputation, and it is increasingly important to address post-amputation pain as long-term function in this younger population of patients with limb loss is highly dependent on a pain-free residual limb [4]. Phantom limb pain (PLP) and residual limb pain (RLP) are potentially disabling sequelae of LEA with estimates of prevalence up to 60% that can have crippling effects on postoperative ambulation rates [5]. With a younger, growing population of patients living with lower extremity limb loss, the burden of chronic post-amputation pain will likely become more consequential.

Patients with LEA are a heterogeneous population, and not all patients follow the same path to amputation. Minor LEAs, defined as distal to the ankle, are widely used as a limb-preserving strategy to maximize function while surgically managing devitalized tissue whenever possible. Though this strategy has benefits, many patients with minor LEAs have systemic disease progression necessitating higher-level amputation. Risk factors associated with post-amputation pain have been proposed, but it is unclear if progressive loss of sensory territory through the foot and ankle may modulate or accentuate the resulting pain when controlling for covariates. For example, serial peripheral nerve transections and repeated episodes of peripheral pain may pre-dispose patients to increased postoperative pain and lead to centralization. Conversely, it is possible that a more gradual loss of sensory territory may allow for improved adaptive cortical plasticity that can mitigate the development of post-amputation pain and serve a protective role by decreasing the rate at which input to the somatosensory cortex is lost. While it has been described that an acute amputation failure necessitating re-amputation or surgical revision is strongly predictive of worse pain outcomes [6,7], there is limited evidence comparing pain outcomes between progressive and primary major LEA while controlling for patient co-morbidities.

In this study, we aim to primarily compare chronic pain outcomes between patients undergoing a minor LEA with progression to a major LEA and patients with primary major LEA. We utilized the TriNetX global healthcare database, which aggregates almost 200 million de-identified patient records [8], to compare rates of chronic post-amputation pain, residual and phantom limb pain, complication rates, narcotic prescribing, secondary procedures, and postoperative mobility. We hypothesized that time-dependent, progressive loss of sensory territory in the lower extremity, not associated with a minor amputation failure, would be associated with lower rates of residual and phantom limb pain.

## 2. Methods

### 2.1. Data Source and Study Design

We conducted a retrospective cohort study using the TriNetX Global Collaborative Network, a federated research network containing de-identified electronic health record data from participating healthcare organizations (HCOs). The study query and analyses were performed July–October 2026. At the time of the query, the Global Collaborative Network included data from 186 HCOs.

The study population included adults aged 18 years or older who underwent major lower-extremity amputation. Major amputations were identified using Current Procedural Terminology (CPT) codes 27590, 27591, 27592, 27880, 27881, and 27882. Minor amputations were identified using CPT codes 28800, 28805, 28810, 28820, and 28825. For both study groups, the index event was the first recorded major amputation meeting the relevant cohort criteria.

### 2.2. Study Population and Cohort Construction

Patients were classified into two cohorts based on their history of minor and major amputation. Cohort A included patients who underwent a minor amputation followed by a subsequent, distinct major amputation. Cohort B included patients who underwent a primary major amputation, defined as having no prior recorded minor amputation based on the prespecified CPT criteria. To examine whether the interval between minor and major amputation was associated with outcomes after major amputation, Cohort A was further sub-divided using six intervals: 0–3 months, 3–6 months, 6–12 months, ≥1 year, ≥2 years, and ≥5 years. The 0–3-month, 3–6-month, and 6–12-month groups represented mutually exclusive intervals. The ≥1-year, ≥2-year, and ≥5-year groups represented minimum elapsed-time thresholds and therefore contained overlapping patients.

### 2.3. Covariates and Propensity Score Matching

Propensity score matching was performed within TriNetX to reduce baseline differences between patients who progressed from minor to major amputation and patients who underwent primary major amputation. Matching was performed separately for each of the six Cohort A definitions. Baseline covariates were identified using all available electronic health record data before the index major amputation, with the consideration period ending one day before the index event. Information or new diagnoses recorded on or after the index event were not included in the propensity score model.

There were 99 variables used to match the cohorts, including age at index, sex, race, ethnicity, and prespecified clinical characteristics. Race was categorized as White, Black or African American, Asian, American Indian or Alaska Native, Native Hawaiian or Other Pacific Islander, or Other Race. Ethnicity was categorized as Hispanic or Latino or not Hispanic or Latino according to TriNetX categories. The exhaustive list of clinical covariates can be seen in Supplemental Table S1, but most importantly, cohorts were matched for preoperative pain, diabetes mellitus, diabetic and other peripheral neuropathies, peripheral vascular disease, atherosclerosis, acute and chronic osteomyelitis, foot ulceration, mood disorders, heart and kidney disease, and prior amputations or prior nerve-based interventions.

Propensity scores were matched in a 1:1 ratio without replacement using a greedy nearest-neighbor algorithm and a caliper width of 0.1 of the pooled standard deviation of the logit of the propensity score. Covariate balance after matching was assessed using standardized mean differences (SMDs), with an absolute SMD < 0.10 considered indicative of satisfactory balance. Separate propensity score-matched analyses were conducted for the primary pain outcomes and secondary outcomes such that separate, simultaneous analyses could be performed either including or excluding patients with documented pre-index (major LEA) diagnoses of the corresponding pain outcome while limiting time-dependent variation in TriNetX data. Meaning, pain was kept distinct to perform a sub-analysis for incident outcomes. All analyses used the same covariates and matching procedures described above.

### 2.4. Outcome Assessment

Outcomes were assessed from one day to five years after the index major amputation for each of the six cohort comparisons. Primary outcomes were residual and phantom limb pain. Secondary outcomes included opioid use and non-narcotic analgesic use (binary measures of the presence or absence of prescriptions, not dosing), stump or amputation revision and associated complications or infection, death, and reduced ambulatory status. Outcomes were identified using ICD-10-CM, CPT, RxNorm, TriNetX generic drug class, and mortality variables, as appropriate. Complete outcome definitions and corresponding codes are provided in Supplemental Table S2. For the primary pain outcomes, two analyses were conducted as stated: one including patients regardless of preexisting pain diagnoses and another excluding patients with a documented diagnosis of the corresponding pain outcome before the index major amputation. The latter analysis was performed to only assess newly documented postoperative pain. Secondary outcomes were assessed without excluding patients with prior documentation of the corresponding outcome; therefore, these results represent occurrence during follow-up rather than exclusively incident events.

### 2.5. Statistical Analysis

All cohort construction, propensity score matching, and outcome analyses were conducted using the TriNetX LIVE (TriNetX LLC, Cambridge, MA, USA) analytics platform. Each of the six minor-to-major amputation intervals was analyzed independently. For each outcome, the number of patients with the outcome was determined in each matched cohort. Between-group comparisons were summarized using risk differences with 95% confidence intervals, z statistics, and corresponding *p* values.

Time-to-event analyses were performed using the Kaplan–Meier method over the same follow-up period. Patients were censored at the earliest of five years after the index event, the end of available follow-up, or their last recorded activity in the electronic health record. For nonfatal outcomes, patients who died before experiencing the outcome of interest were censored at death; competing-risk methods were not used. Kaplan–Meier analyses were summarized using hazard ratios with 95% confidence intervals and corresponding *p* values. Because each time interval was constructed and matched separately, effect estimates from the 0–3-month, 3–6-month, 6–12-month, ≥1-year, ≥2-year, and ≥5-year analyses represent separate matched comparisons. No adjustment was made for multiple comparisons. All statistical tests were two-sided, and *p* < 0.05 was considered statistically significant.

## 3. Results

Before propensity score matching, Cohort A included 6732 patients in the 0–3-month analysis, 2450 in the 3–6-month analysis, 2262 in the 6–12-month analysis, 4789 in the ≥1-year analysis 3259 in the ≥2-year analysis, and 1162 in the ≥5-year analysis. Cohort B included 44,080 patients before matching.

### 3.1. Cohort for Analysis

**Figure 1:**
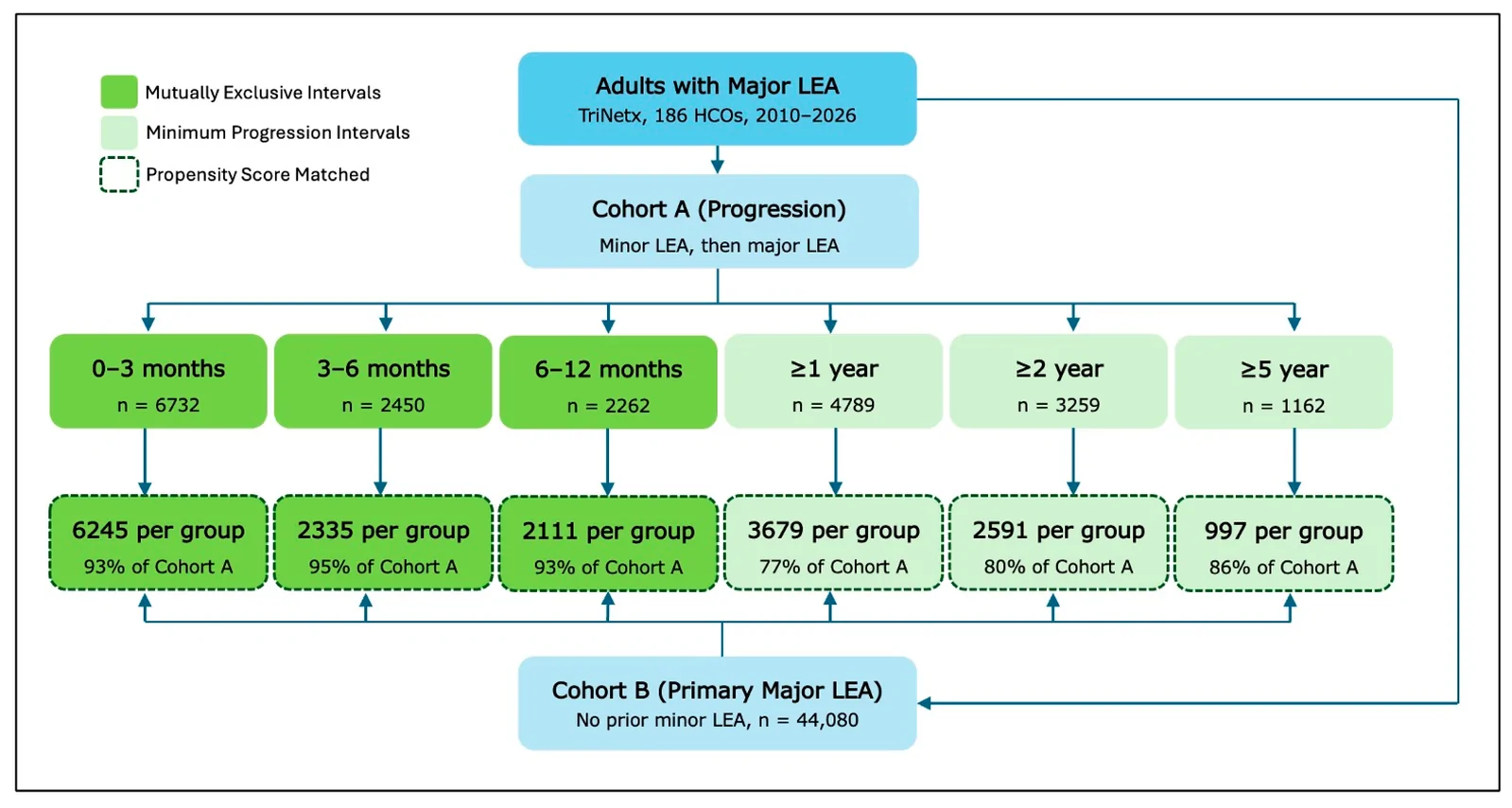
The flowchart for cohort creation where the lower row of green boxes indicates the matched cohorts.

**Figure 2:**
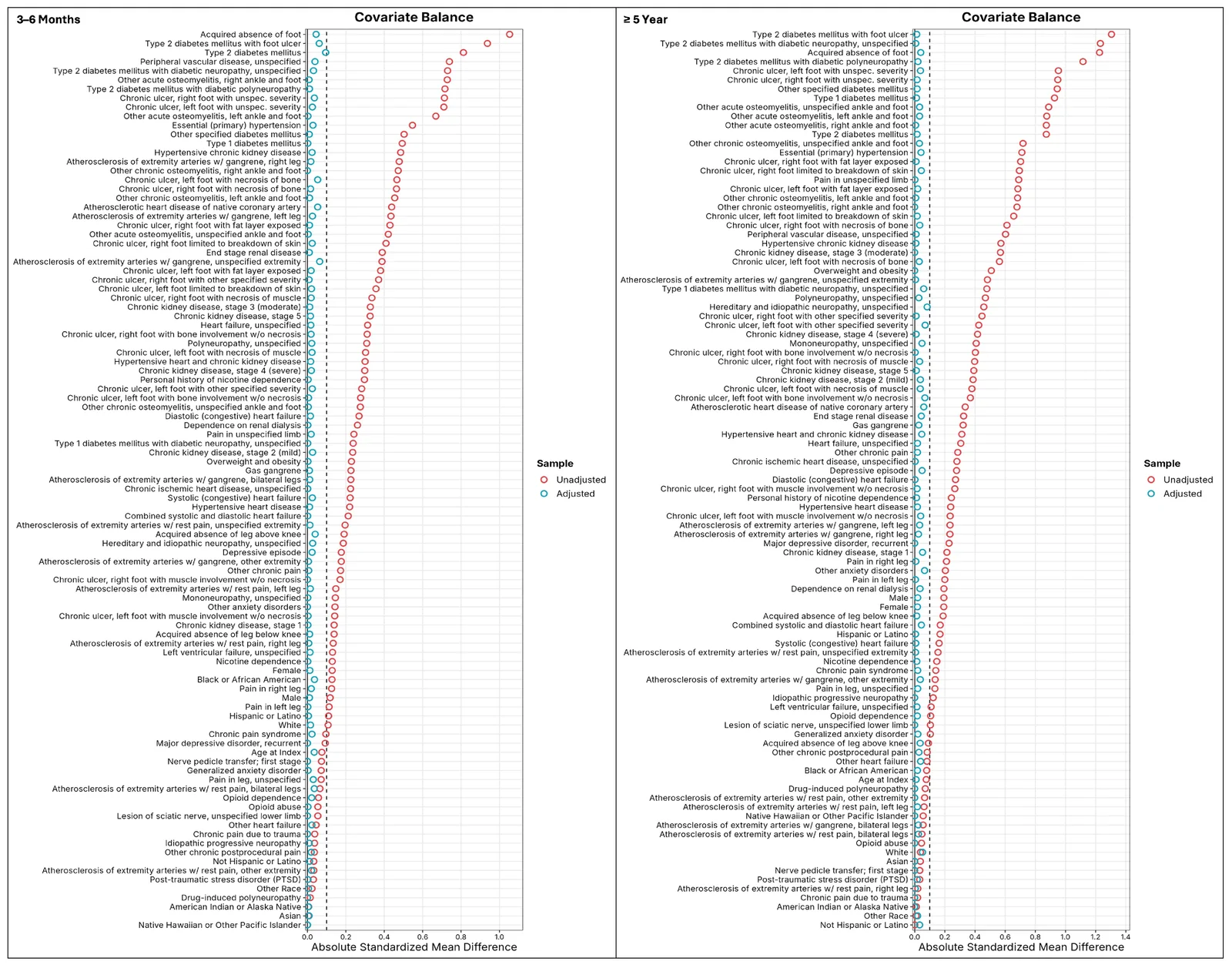
The two SMD tables with the least well-matched covariates on average. The set caliper at 0.1 is indicated by vertical dotted line. The 3–6-month interval had an average pre-match SMD of 0.276 that decreased to 0.016 after matching. The 5-year interval had an average pre-match SMD of 0.369 that decreased to 0.022 after matching.

Six cohorts were created within the progression group as described after 1:1 propensity score matching with a corresponding matched sample of primary major amputation patients in Cohort B. Matched cohorts comprised 6245 patients per group for the 0–3-month interval (92.8% of the unmatched cohort), 2335 per group for the 3–6-month interval (95.3% of the unmatched cohort), 2111 per group for the 6–12-month interval (93.3% of the unmatched cohort), 3679 per group for the ≥1-year interval (76.8% of the unmatched cohort), 2591 per group for the ≥2-year interval (79.5% of the unmatched cohort), and 997 per group for the ≥5-year interval (85.8% of the unmatched cohort). Absolute standardized mean differences (SMDs) were on average less than 0.10 after matching for covariates, indicating adequate covariate balance between the two groups. Figure 1 presents the flowchart for cohort creation with sample sizes before and after matching while Figure 2 presents the SMD values for the two least well-matched progression intervals with all covariates still within the set caliper. The remaining SMD values are presented in Supplemental Figure S1.

### 3.2. Residual Limb Pain

**Figure 3:**
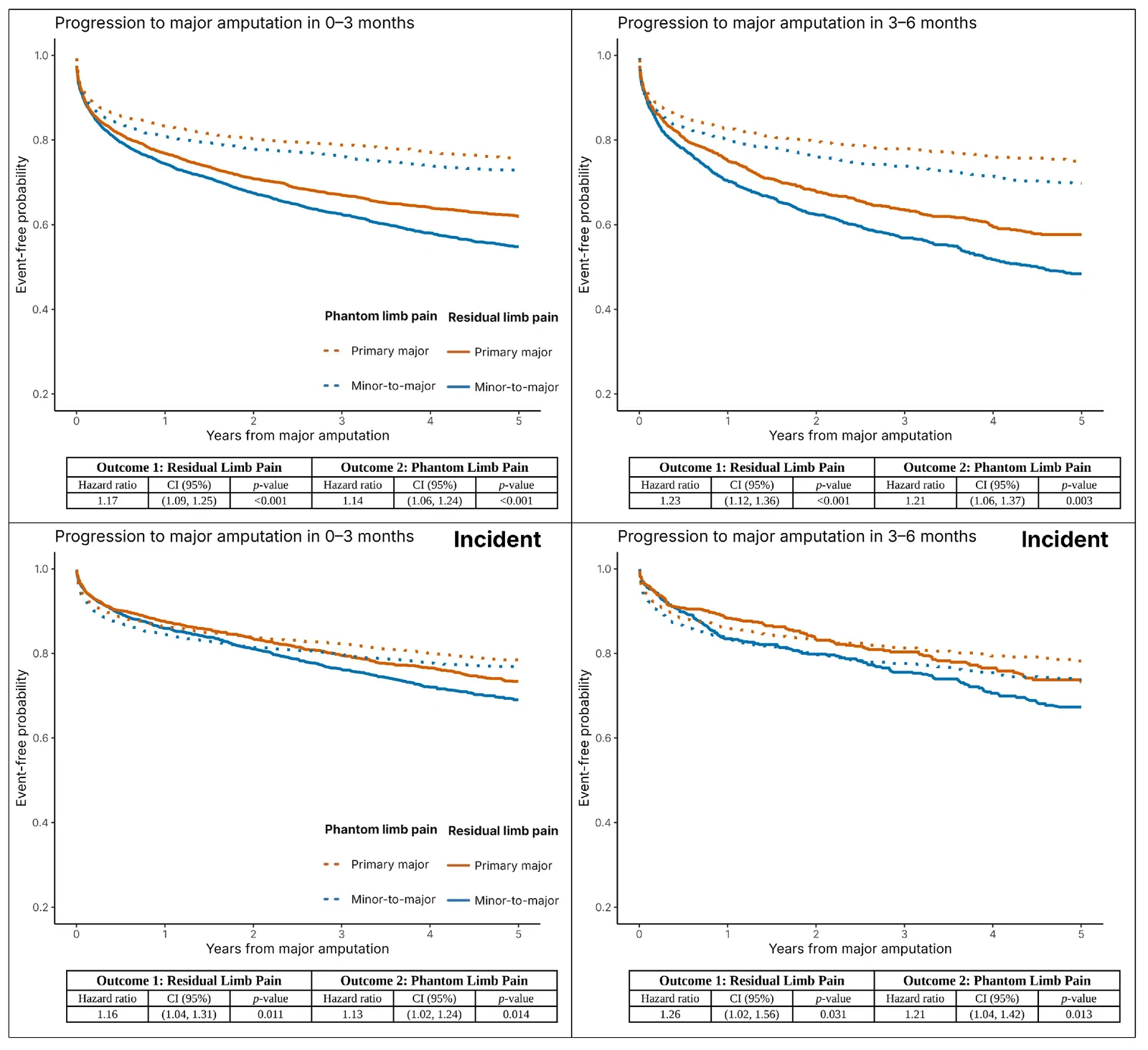
Kaplan–Meier plots and associated statistics for the primary outcomes if progression occurs at 0–3 months or 3–6 months with exclusive, incident outcomes presented in the lower panels.

**Figure 4:**
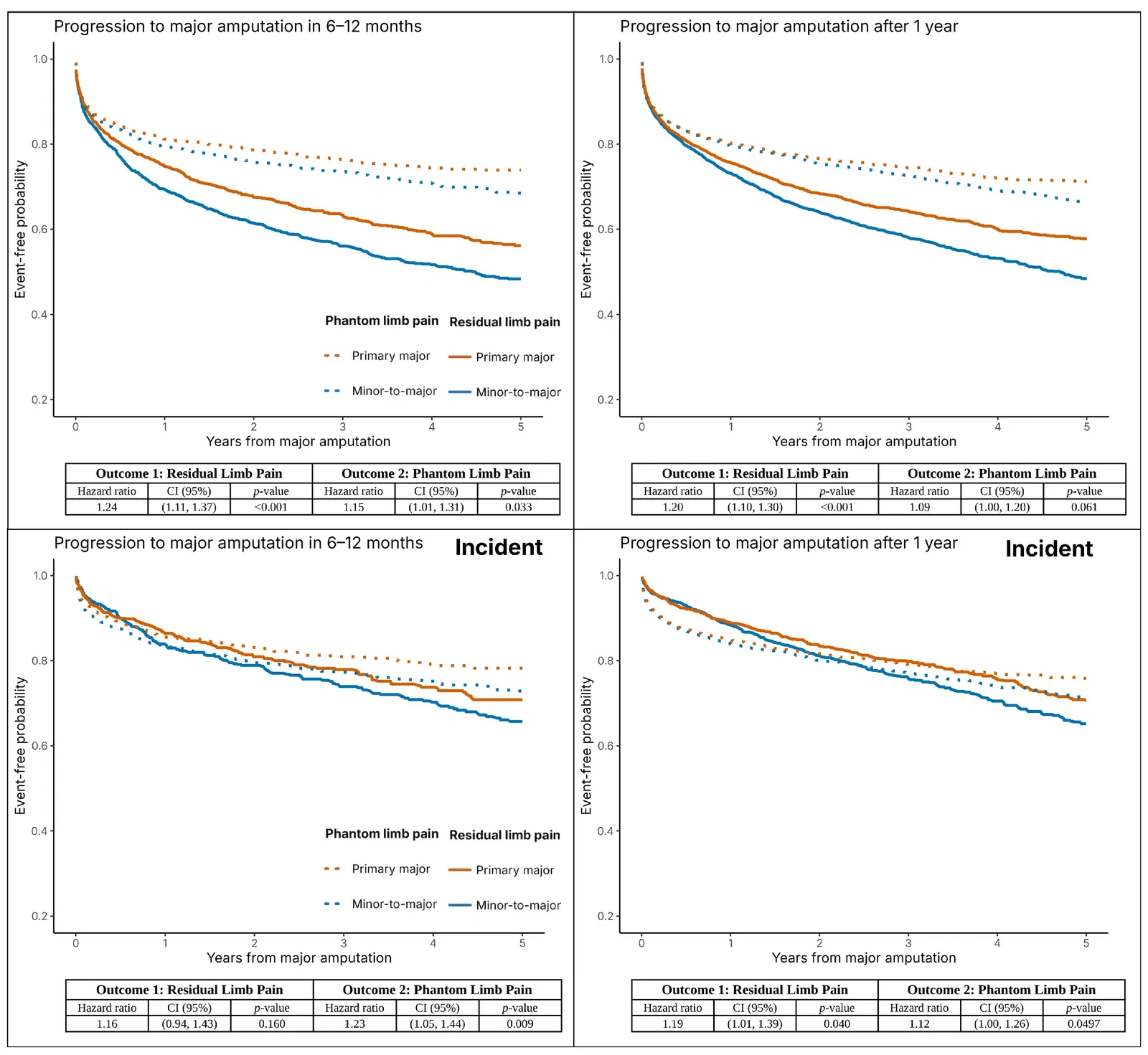
Kaplan–Meier plots and associated statistics for the primary outcomes if progression occurs at 6–12 months or ≥1 year with exclusive, incident outcomes presented in the lower panels.

**Figure 5:**
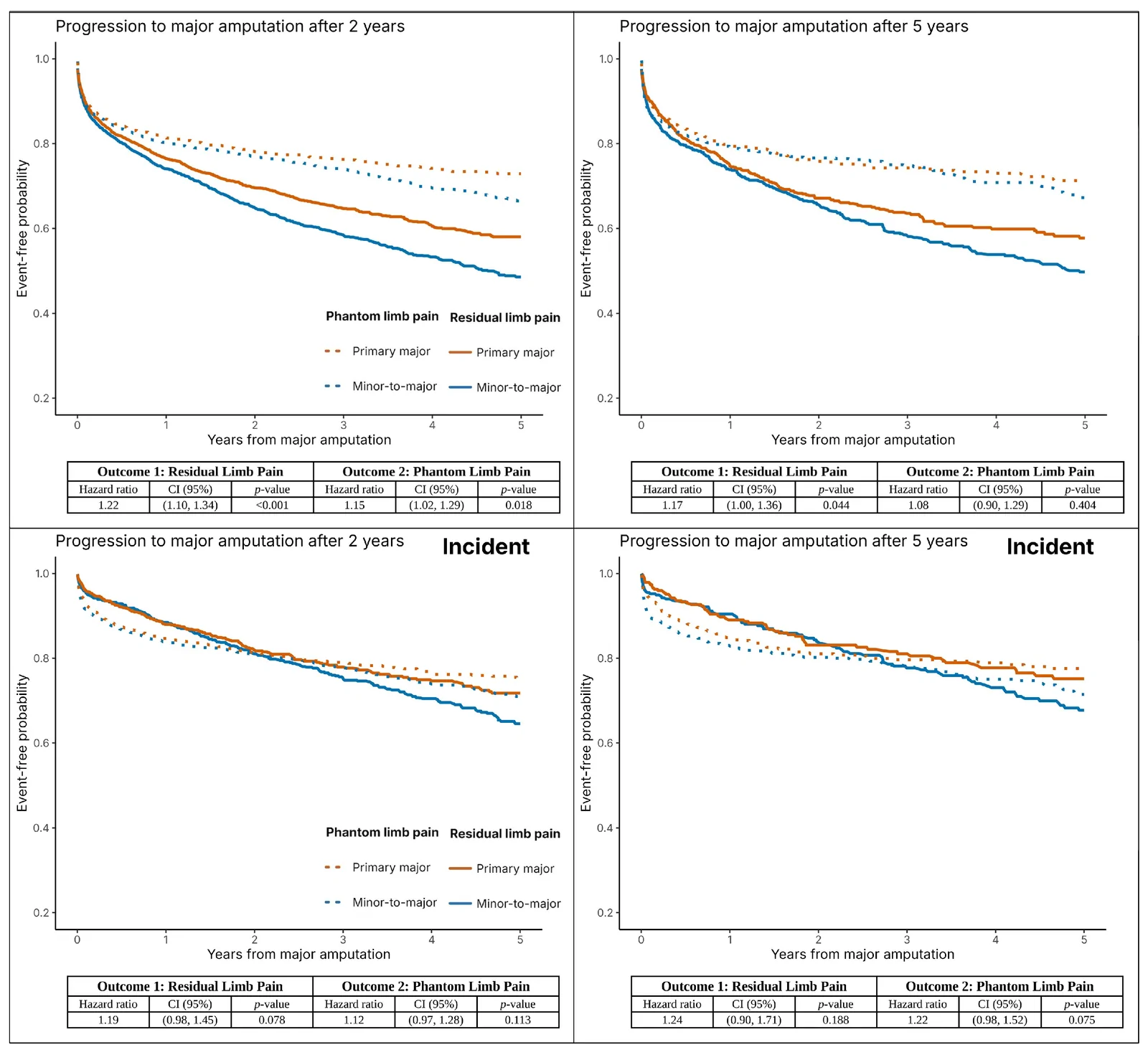
Kaplan–Meier plots and associated statistics for the primary outcomes if progression occurs at ≥2 years or ≥5 years with exclusive, incident outcomes presented in the lower panels.

Patients who progressed from minor to major LEA had higher risk of RLP compared to primary major LEA across all time intervals (Table 1). Absolute risks of RLP for Cohort A ranged from 31.6% to 38.3%, compared to 26.0% to 30.5% in Cohort B (*p* ≤ 0.0001 for all comparisons except ≥5-year interval *p* = 0.005), with the 6–12-month group having the greatest risk difference of 8.5% (Table 1). Interestingly, risk differences begin to decline when progression occurs ≥ 1 year from the minor amputation with the smallest, but still significantly different, risk difference at 5 years between amputations. However, these differences are primarily due to the risk of Cohort B increasing through the time intervals while the risk for Cohort A stays approximately the same, with an average of 36.0% ± 2.3% (standard deviation) across all intervals examined. Time-to-event analysis was consistent with these trends, with hazard ratios (HRs) showing earlier onset of residual limb pain for the 0–3 months, 3–6 months, 6–12 months, ≥1 year, and ≥2 years groups (all log-rank *p* < 0.001) (Figure 3, Figure 4 and Figure 5). The ≥5-year interval was also significant, though with the weakest effect (HR 1.17 [1.00–1.36]; log-rank *p* = 0.044) (Table 1).

For incident outcomes (Table 2, Figure 3, Figure 4 and Figure 5), sample sizes decrease significantly, and absolute risk for RLP is on average 13.0% lower for the progression cohorts compared to non-incident outcomes. Risk difference increases very mildly with increasing intervals while all intervals have highly significant differences. Very similar hazard ratios can be appreciated through Figure 3, Figure 4 and Figure 5 comparing the upper and lower panels while confidence intervals widen and significant values decrease potentially secondary to lost sample sizes.

### 3.3. Phantom Limb Pain

Phantom limb pain (PLP) was more prevalent in Cohort A, with absolute risks ranging from 20.8% to 25.0% compared to 17.6% to 22.3% in Cohort B (Table 1). These differences were statistically significant at all time intervals (*p* < 0.001 for all comparisons) except for the ≥5-year interval (24.9% vs. 22.3%, *p* = 0.17). Interestingly, risk for developing phantom limb pain after progression appears to increase after the first 3 months and then stabilize, from 20.8% at 0–3 months to 24.5–25.0% at ≥1 year, albeit with a small difference of about 4%. Kaplan–Meier analysis demonstrated significantly increased hazard for Cohort A at the 0–3-month (HR 1.14 [1.06–1.24]), 3–6-month (HR 1.21 [1.06–1.37]), 6–12-month (HR 1.15 [1.01–1.31]), and ≥2-year (HR 1.15 [1.02–1.29]) intervals, with nonsignificant trends at ≥1 year (HR 1.09 [1.00–1.20]; *p* = 0.061) and ≥5 years (HR 1.08 [0.90–1.29]; *p* = 0.40) (Figure 3, Figure 4 and Figure 5).

For incident outcomes (Table 2, Figure 3, Figure 4 and Figure 5), sample sizes decrease much less than for RLP, and absolute risk for PLP is on average 3.7% lower for the progression cohorts compared to non-incident outcomes. All intervals have significance in the risk differences although after the 0–3-month interval, these values are roughly stable and small. Hazard ratios are again largely unchanged with the additional exclusivity screening of the incident analysis while only one significance value was lost—the ≥2-year interval.

### 3.4. Prescription Pain Medication Use

Both cohorts had high rates (>90%) of opioid prescribing, with moderately lower rates in Cohort A at the 0–3-month, 6–12-month, ≥1-year, ≥2-year, and ≥5-year intervals, though all risk differences were less than 3.1% (Table 3). For time-to-event analysis, there was lower hazard of opioid prescribing in Cohort A at all time intervals (HRs ranging from 0.82 to 0.92; all log-rank *p* ≤ 0.003) (Supplemental Table S3).

Rates of non-narcotic analgesic use ranged from 70 to 80% for both cohorts, with the only significant differences noted at the 0–3-month and 3–6-month time intervals during which Cohort A had moderately higher use (73.7% vs. 71.5% for 0–3 months, *p* = 0.007; 80.0% vs. 76.3% for 3–6 months, *p* = 0.004) (Table 3). Kaplan–Meier analysis was similar and mostly nonsignificant, with a lower hazard ratio for Cohort A at the ≥2-year interval (HR 0.90 [0.84–0.96], log-rank *p* = 0.001) (Supplemental Table S3).

### 3.5. Amputation Revision or Complications

**Figure 6:**
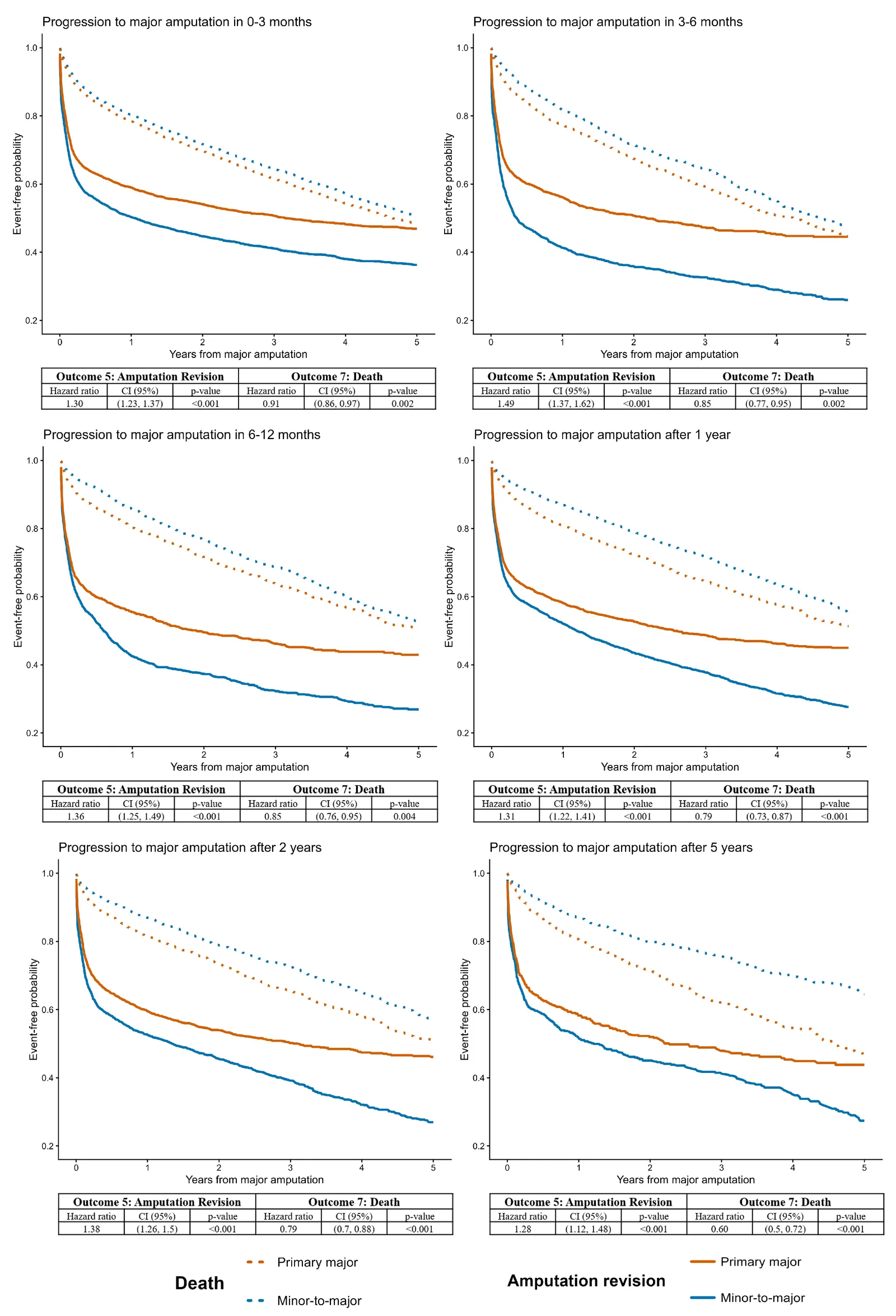
Kaplan–Meier plots and associated statistics for mortality risk and amputation revision or complication risk.

When comparing rates of amputation revision and complications, there were consistent differences at all time intervals, with significantly higher rates in Cohort A (all *p* < 0.0001) (Table 4). Absolute risk for Cohort A ranged from 52.7% to 62.5% versus 41.8% to 46.1% in Cohort B, with the greatest difference at the 3–6-month interval (62.5% vs. 45.2%). Hazard ratios were also significantly higher for all time intervals (all log-rank *p* < 0.001) (Figure 6).

### 3.6. Reduced Ambulatory Status

Patients in Cohort A were more likely to experience reduced ambulatory status in the earlier time interval groups, with absolute risk differences of +2.0% for 0–3 months (*p* = 0.010), +4.2% for 3–6 months (*p* = 0.003), +5.3% for 6–12 months (*p* < 0.001), and +4.6% for ≥1 year (*p* < 0.001) (Table 4). Time-to-event analysis was not statistically significant at any time interval (Supplemental Table S3).

### 3.7. Death

Cumulative mortality rates were lower in Cohort A for the ≥1 year (30.3% vs. 32.7%, *p* = 0.04), ≥2 years (29.9% vs. 32.9%, *p* = 0.04), and ≥5 years (25.7% vs. 36.6%, risk difference, *p* < 0.001) time intervals while no difference was found at earlier progression time points (Table 3). Time-to-event analysis showed moderately lower hazard of death in Cohort A for all time intervals, with effect sizes increasing in magnitude over time from 0.91 [0.86–0.97] for 0–3 months to 0.60 [0.50–0.72] for ≥5 years (all log-rank *p* ≤ 0.004) (Figure 6).

## 4. Discussion

In this strict, 99 covariate, propensity score-matched analysis, minor-to-major progression of lower-extremity amputation along clinically relevant time points can be appreciated to have significantly worse RLP and PLP outcomes, as well as increased rates of amputation revision and complications. Risk differences for RLP range from 4.3 to 8.5% and range from 10.9 to 17.2% for amputation revisions or complications if progression occurs within one year of the minor amputation. The risk for developing either outcome declines slightly with increasing intervals between amputations, although it remains significantly worse for progression cohorts. Interestingly, in contrast to our hypothesis, PLP absolute risk demonstrates the opposite trend as it increases with increasing time intervals between minor and major amputations while risk differences demonstrate little to no time dependence. The incident pain outcomes represent a clinically distinct population after minor LEA—patients who achieve a pain-free state vs. those that develop subsequent chronic pain. This analysis reveals a moderate protective factor in RLP outcomes of 13.0% on average with little effect (~3%) on PLP outcomes. These trends are highly informative for patient counseling and may inform providers of earlier opportunities to intervene in patients’ surgical treatment for limb loss.

While risk factors have been associated with post-amputation pain, such as proximal amputation level, psychiatric history, and younger age [9,10], the prior literature has never examined the time interval between amputations for resulting pain outcomes. To accomplish this, we intentionally chose clinically relevant time points for amputation progression. Approximately 17–59% of patients with a LEA secondary to diabetes or peripheral arterial disease will progress to a major amputation within 5 years [11,12] while the median time to re-amputation is 8–14 months [13]. Additionally, a 2022 study found worse pain outcomes in failed transmetatarsal amputations that warranted progression [7]. We therefore chose our progression intervals to correlate with these clinical trajectories, where the 0–3-month interval correlates with amputation failure, and longer intervals correlate with successful limb salvage with underlying disease progression to determine the effects surgical timing has on pain outcomes.

This is also the reasoning behind why the shorter progression intervals are bounded while the ≥1-year intervals have no upper bound. Patients with rapid progression represent vastly different clinical scenarios that can readily influence outcomes compared to patients with progression ≥1 year from the minor LEA where we are primarily interested in controlling for the minimum interval between surgical interventions. The incident analysis for the primary outcomes was kept distinct to also correlate with meaningful clinical trajectories where patients either do or do not return to a pain-free state prior to progression to a major LEA. We believe this represents two distinct clinical presentations prior to the major LEA that warranted distinct analyses and revealed that RLP decreases much more than PLP if a pain-free state is achieved prior to the major LEA. This may also indicate that the major amputation is the primary driver of developing PLP compared to minor amputation.

Before further conclusions are drawn from the primary outcomes, we believe the observed trend in the death statistics strongly support the validity of our analysis, clinical relevance and applicability, and the reliability of coding consistency between cohorts. We used TriNetX’s default matching algorithm—greedy nearest-neighbor matching with a caliper width of 0.1 pooled standard deviations of the logit of the propensity score. This is more strict than necessary and was most likely successful in this setting given Cohort B was more than five times larger than the largest interval for Cohort A, allowing such strict matching. A widely cited article from 2010 simulated an observational study to determine that an optimal caliper width of 0.2 eliminated at least 98% of bias in the estimator [14], and Trinetx’s 0.1 caliper is more strict.

While a secondary outcome, differences in survival remain crucial to appreciate between cohorts. The trend demonstrates no difference in survival if progression occurs within one year of the minor amputation with an increasingly protective effect with increasing time intervals between amputations. With greater than five years between amputations, the progression cohort demonstrates a 10.9% decreased risk of mortality that is readily apparent on the Kaplan–Meier plots in Figure 6, where all differences persist. This correlates well with clinically known vascular or diabetic complications, or primary disease progression, that most frequently result in amputation progression in the US population. Therefore, patients with longer time intervals between amputation progression most likely have both less-severe disease at the time of minor amputation and slower disease progression warranting the secondary, major LEA. Conversely, if strictly matched, co-morbid patients are treated with primary major LEA without a preceding minor LEA, their underlying diagnoses can be inferred to be further progressed and therefore worsen mortality rates. This trend is also readily apparent in Table 4, where no significant difference is found in mortality until the ≥1 year interval, after which, increasing differences and significance values can be appreciated.

We must also recognize and acknowledge the survivorship bias inherent in defining our progression cohorts by large time intervals such as ≥2 and ≥5 years. Beyond differences in underlying disease severity and primary disease progression, the patients in these cohorts must have survived for 2–5 years after the minor amputation prior to being compared for outcome analysis to patients treated up front with a major LEA. This may self-select patients with healthier lifestyle habits, closer medical follow-up, higher medical literacy, more consistent medication use, or a combination thereof which results in increased survival independent of surgical interventions. However, because these treatment pathways are solely defined on time intervals, this data would still be clinically relevant for counseling and risk stratification for patients whose treatment falls within these intervals.

While postoperative nerve pain remains incredibly complex, it is well accepted that the mechanisms underlying RLP and PLP are distinct. RLP is primarily a peripheral etiology involving the residual limb soft tissues, bony stump, and transected peripheral nerves while PLP is a far more complex, poorly understood interplay between the transected peripheral nerves, spinal circuits, and supratentorial processing. This results in either adaptive or maladaptive changes to cortical processing of lost somatosensory input and motor control of the amputated limb [15,16,17,18,19]. It is reasonable to therefore expect differences in operative risk factors for these types of post-amputation pain, where repeated peripheral nerve transections, bony shortening and soft tissue re-draping can be expected to worsen RLP outcomes as Table 1 and Table 2 and Figure 3, Figure 4 and Figure 5 demonstrate through comparing Cohort A to Cohort B, independent of time intervals.

It is also reasonable to expect increasing time intervals between amputations to be protective for RLP; however, interestingly, the trend in risk differences in Table 1 are also driven by changes in risk for Cohort B, rather than absolute decreases in risk for Cohort A. Even more stark is the difference in risk for Cohort B between Table 1 and Table 2 with the incident analysis which is on average 11.8% lower. This strongly indicates that there may be underlying differences in patient factors that are more influential on pain outcomes than surgical timing between amputations. Unfortunately, within the TriNetX software, relative frequencies of matched covariates cannot be examined after outcome analyses are run. Risk in Cohort A between Table 1 and Table 2 can also be appreciated to decrease. This may indicate that the same underlying reasons for these progression patients returning to a pain-free state after their minor amputation are also moderately protective after the major amputation and warrant further investigation.

On the other hand, our analysis revealed an opposing clinical paradigm for PLP. The absolute risk for developing PLP can be seen to steadily increase for Cohort A in both Table 1 and Table 2, though the risk for Cohort B also rises, so risk differences do not widen. The trend is not apparent on Kaplan–Meier analysis, where the hazard ratio was greatest at the 3–6-month interval and nonsignificant at the ≥1-year and ≥5-year intervals. We hypothesized that PLP would decrease with increasing intervals of amputation progression as this would decrease the rate at which somatosensory input was lost, potentially allowing patients to more gradually adjust to limb loss. However, the opposite trend was discovered for progression patients with very little time dependence on risk differences. Pulling from the largest body of plasticity—stroke—there is evidence that recurrent central insult worsens neurological recovery [20]. There are also animal models of limb loss that demonstrate dysregulation of central neurotransmitters, leading to increased excitation and decreased inhibition in the deprived somatosensory cortex [21].

Extrapolating to clinical patients with limb loss, this data may indicate that the resulting adaptive or maladaptive processes that occur following the major amputation are the most important and crucial to target as they most significantly influence the patient’s final outcome, while appreciating that longer intervals between progression may increase risk for developing PLP. There are unfortunately no current clinical pathways for nerve-based intervention after minor LEA, and it remains unknown if optimizing pain outcomes after the minor LEA would result in improved outcomes after the major LEA as our incident subanalysis would suggest. This concept could be leveraged as an ideal potential target for phantom motor imagery or execution which have primarily been used after major amputation.

We recognize that this study is not without limitations. The TriNetX database is reliant on correct coding from participating institutions and correct timing of entries, and ICD-10 codes are not reflective of disease severity. Furthermore, pain cannot be fully characterized through ICD10 codes alone or be used to differentiate between peripheral and central pathology, and may be coded differently by different providers. We unfortunately also found a global lack of neuroma coding that obviated the ability to complete an analysis on true neurogenic pain. These codes are likely more commonly used by specialists rather than routine providers caring for patients with limb loss. Despite matching on a large number of covariates with strict calipers, we can also not account for implicit decision-making bias in how providers determine the optimal initial level for lower-extremity amputation, at what point to progress to a higher level, or when an arbitrary threshold for delayed wound healing has been reached warranting a more proximal amputation. As previously stated, sample sizes are also limited for the longer time intervals, and relative frequencies of covariates cannot be examined after analyses are performed to more fully attribute differences to surgical timing and not underlying differences in diagnoses. Finally, because multiple outcomes were evaluated across several temporal intervals without adjustment for multiple comparisons, the possibility of type I error should be considered when interpreting statistically significant findings, particularly those with *p* values near the significance threshold. These results should therefore be interpreted cautiously.

## 5. Conclusions

The risk for developing residual limb pain is most significant if progression occurs 3–12 months after minor amputation and amputation complications if progression occurs within one year, while the risk for developing PLP increases with increasing intervals. While highly informative for patient counseling and for providers to potentially incorporate pain adjuncts in clinical care pathways, this data indicates that patients’ path to major LEA heavily influences their pain and surgical outcomes and must be accounted for.

## Figures and Tables

**Table 1:** Cumulative risk and associated statistics for the primary outcomes without excluding patients for pre-index amputation ICD10 codes that correspond to the outcomes.

**Outcome 1: Residual Limb Pain**								
**Time Interval**	**Cohort A**		**Cohort B**		**Risk Difference**	**CI (95%)**	**z**	***p* Value**
	**Sample with Outcome**	**Risk**	**Sample with Outcome**	**Risk**				
0–3 months	1971	31.56%	1626	26.04%	5.52%	(3.94%, 7.11%)	6.817	<0.0001
3–6 months	855	36.62%	672	28.78%	7.84%	(5.16%, 10.52%)	5.708	<0.0001
6–12 months	808	38.28%	629	29.80%	8.48%	(5.63%, 11.33%)	5.814	<0.0001
1+ years	1347	36.61%	1083	29.44%	7.18%	(5.03%, 9.32%)	6.544	<0.0001
2+ years	943	36.40%	749	28.91%	7.49%	(4.94%, 10.03%)	5.747	<0.0001
5+ years	363	36.41%	304	30.49%	5.92%	(1.78%, 10.05%)	2.8	0.0051
**Outcome 2: Phantom Limb Pain**								
**Time Interval**	**Cohort A**		**Cohort B**		**Risk Difference**	**CI (95%)**	**z**	***p* Value**
	**Sample with Outcome**	**Risk**	**Sample with Outcome**	**Risk**				
0–3 months	1300	20.82%	1099	17.60%	3.22%	(1.84%, 4.60%)	4.566	<0.0001
3–6 months	542	23.21%	430	18.42%	4.80%	(2.47%, 7.12%)	4.037	<0.0001
6–12 months	510	24.16%	419	19.85%	4.31%	(1.81%, 6.81%)	3.381	0.0007
1+ years	920	25.01%	799	21.72%	3.29%	(1.36%, 5.22%)	3.334	0.0009
2+ years	636	24.55%	530	20.46%	4.09%	(1.82%, 6.36%)	3.526	0.0004
5+ years	248	24.87%	222	22.27%	2.61%	(−1.12%, 6.33%)	1.372	0.1701

**Table 2:** Cumulative risk and associated statistics for the primary outcomes as incident.

**Outcome 1: Residual Limb Pain—Incident**								
**Time Interval**	**Cohort A**		**Cohort B**		**Risk Difference**	**CI (95%)**	**z**	***p* Value**
	**Sample with Outcome**	**Risk**	**Sample with Outcome**	**Risk**				
0–3 months	624	20.49%	521	16.20%	4.29%	(2.37%, 6.21%)	4.391	<0.0001
3–6 months	190	21.57%	153	15.84%	5.73%	(2.17%, 9.29%)	3.162	0.0016
6–12 months	190	23.90%	162	18.20%	5.70%	(1.80%, 9.60%)	2.872	0.0041
1+ years	318	23.45%	270	17.26%	6.19%	(3.26%, 9.12%)	4.158	<0.0001
2+ years	220	24.50%	184	17.81%	6.69%	(3.03%, 10.34%)	3.603	0.0003
5+ years	85	23.88%	66	17.14%	6.73%	(0.92%, 12.55%)	2.273	0.023
**Outcome 2: Phantom Limb Pain—Incident**								
**Time Interval**	**Cohort A**		**Cohort B**		**Risk Difference**	**CI (95%)**	**z**	***p* Value**
	**Sample with Outcome**	**Risk**	**Sample with Outcome**	**Risk**				
0–3 months	928	17.43%	783	14.81%	2.62%	(1.22%, 4.02%)	3.67	0.0002
3–6 months	380	19.65%	284	15.09%	4.56%	(2.16%, 6.96%)	3.713	0.0002
6–12 months	362	20.22%	268	15.32%	4.90%	(2.39%, 7.41%)	3.81	0.0001
1+ years	639	20.61%	532	17.30%	3.31%	(1.35%, 5.26%)	3.313	0.0009
2+ years	452	20.78%	376	17.58%	3.20%	(0.86%, 5.55%)	2.671	0.0076
5+ years	181	21.37%	142	16.99%	4.38%	(0.63%, 8.14%)	2.283	0.0224

**Table 3:** Cumulative risk and associated statistics for prescription medication use.

**Outcome 3: Opioid Use**								
**Time Interval**	**Cohort A**		**Cohort B**		**Risk Difference**	**CI (95%)**	**z**	***p* Value**
	**Sample with Outcome**	**Risk**	**Sample with Outcome**	**Risk**				
0–3 months	5457	90.32%	5587	92.47%	−2.15%	(−3.15%, −1.15%)	−4.217	<0.0001
3–6 months	1928	93.28%	1941	93.90%	−0.62%	(−2.12%, 0.86%)	−0.825	0.41
6–12 months	1726	92.95%	1761	94.83%	−1.89%	(−3.42%, −0.35%)	−2.397	0.0165
1+ years	2968	92.98%	3026	94.80%	−1.81%	(−3.00%, −0.64%)	−3.031	0.0024
2+ years	1858	92.48%	1900	94.57%	−2.09%	(−3.61%, −0.57%)	−2.693	0.0071
5+ years	683	90.95%	706	94.01%	−3.06%	(−5.73%, −0.40%)	−2.25	0.025
**Outcome 4: Non-Narcotic Analgesic Use**								
**Time Interval**	**Cohort A**		**Cohort B**		**Risk Difference**	**CI (95%)**	**z**	***p* Value**
	**Sample with Outcome**	**Risk**	**Sample with Outcome**	**Risk**				
0–3 months	4450	73.65%	4317	71.45%	2.20%	(0.61%, 3.79%)	2.711	0.0067
3–6 months	1654	80.02%	1578	76.34%	3.68%	(1.16%, 6.19%)	2.862	0.0042
6–12 months	1475	79.43%	1429	76.95%	2.48%	(−0.18%, 5.13%)	1.828	0.068
1+ years	2494	78.13%	2485	77.85%	0.28%	(−1.75%, 2.32%)	0.272	0.79
2+ years	1550	77.15%	1594	79.34%	−2.19%	(−4.74%, 0.36%)	−1.683	0.09
5+ years	574	76.43%	583	77.63%	−1.20%	(−5.45%, 3.06%)	−0.552	0.58

**Table 4:** Cumulative risk and associated statistics for the remaining outcomes.

**Outcome 5: Amputation Revision/Complications**								
**Time Interval**	**Cohort A**		**Cohort B**		**Risk Difference**	**CI (95%)**	**z**	***p* Value**
	**Sample with Outcome**	**Risk**	**Sample with Outcome**	**Risk**				
0–3 months	3182	52.66%	2525	41.79%	10.87%	(9.10%, 12.64%)	11.972	<0.0001
3–6 months	1291	62.46%	935	45.23%	17.22%	(14.23%, 20.22%)	11.107	<0.0001
6–12 months	1135	61.12%	857	46.15%	14.97%	(11.80%, 18.14%)	9.148	<0.0001
1+ years	1827	57.24%	1415	44.33%	12.91%	(10.48%, 15.34%)	10.314	<0.0001
2+ years	1148	57.14%	868	43.21%	13.94%	(10.88%, 17.00%)	8.835	<0.0001
5+ years	433	57.66%	343	45.67%	11.98%	(6.97%, 17.00%)	4.647	<0.0001
**Outcome 6: Reduced Ambulatory Status**								
**Time Interval**	**Cohort A**		**Cohort B**		**Risk Difference**	**CI (95%)**	**z**	***p* Value**
	**Sample with Outcome**	**Risk**	**Sample with Outcome**	**Risk**				
0–3 months	1503	24.88%	1383	22.89%	1.99%	(0.47%, 3.51%)	2.560	0.0105
3–6 months	603	29.17%	517	25.01%	4.16%	(1.45%, 6.87%)	3.010	0.0026
6–12 months	559	30.10%	461	24.82%	5.28%	(2.41%, 8.14%)	3.603	0.0003
1+ years	940	29.45%	794	24.87%	4.57%	(2.39%, 6.75%)	4.108	<0.0001
2+ years	635	31.61%	583	29.02%	2.59%	(−0.25%, 5.43%)	1.785	0.0743
5+ years	241	32.09%	226	30.09%	2.00%	(−2.68%, 6.68%)	0.836	0.4031
**Outcome 7: Death**								
**Time Interval**	**Cohort A**		**Cohort B**		**Risk Difference**	**CI (95%)**	**z**	***p* Value**
	**Sample with Outcome**	**Risk**	**Sample with Outcome**	**Risk**				
0–3 months	2119	35.07%	2072	34.29%	0.78%	(−0.92%, 2.48%)	0.898	0.3690
3–6 months	756	36.57%	760	36.77%	−0.19%	(−3.13%, 2.74%)	−0.129	0.8973
6–12 months	608	32.74%	608	32.74%	0.00%	(−3.02%, 3.02%)	0.000	1.0000
1+ years	968	30.33%	1044	32.71%	−2.38%	(−4.66%, −0.10%)	−2.047	0.0406
2+ years	600	29.87%	661	32.90%	−3.04%	(−5.90%, −0.17%)	−2.074	0.0381
5+ years	193	25.70%	275	36.62%	−10.92%	(−15.57%, −6.27%)	−4.568	<0.0001

## Data Availability

All data can be made available on reasonable request to the corresponding author.

## Supplementary Materials

The following supporting information can be downloaded at https://www.mdpi.com/article/10.3390/jcm15218393/s1, Table S1: The 99 covariates used for propensity score matching, Table S2: ICD and CPT codes used for outcome analyses, Table S3: Kaplan–-Meier statistics for the three remaining outcomes that were not plotted in prior figures; Figure S1: The remaining SMD tables are presented. The set caliper at 0.1 is indicated by the vertical dotted line.
