# Supplementary material for: Chronic Pain and Residual Limb Outcomes Following Primary Major Lower Extremity Amputation vs. Minor-to-Major Amputation Progression: A Propensity Score-Matched Analysis
Source: J Clin Med. 2026 Oct 28;15(21):8393. doi: 10.3390/jcm15218393 (PMC13764492; doi:10.3390/jcm15218393)
Supplement: Supplementary file 1 [file jcm-15-08393-s001.zip › jcm-4567597-supplementary.pdf]

Supplemental Table S1. The 99 covariates used for propensity score matching.

Code	Description
**Demographic variables and categories**	
AI	Age at Index
M	Male
F	Female
2186-5	Not Hispanic or Latino
2135-2	Hispanic or Latino
2106-3	White
2054-5	Black or African American
2131-1	Other Race
2028-9	Asian
1002-5	American Indian or Alaska Native
2076-8	Native Hawaiian or Other Pacific Islander
**Clinical variables (ICD-10-CM)**	
G89.29	Other chronic pain
G89.28	Other chronic postprocedural pain
G89.21	Chronic pain due to trauma
G89.4	Chronic pain syndrome
M79.606	Pain in leg, unspecified
M79.604	Pain in right leg
M79.605	Pain in left leg
M79.609	Pain in unspecified limb
E10.40	Type 1 diabetes mellitus with diabetic neuropathy, unspecified
E11.40	Type 2 diabetes mellitus with diabetic neuropathy, unspecified
E11.42	Type 2 diabetes mellitus with diabetic polyneuropathy
G60.3	Idiopathic progressive neuropathy
G60.9	Hereditary and idiopathic neuropathy, unspecified
I70.221	Atherosclerosis of native arteries of extremities with rest pain, right leg
I70.223	Atherosclerosis of native arteries of extremities with rest pain, bilateral legs
I70.228	Atherosclerosis of native arteries of extremities with rest pain, other extremity
I70.222	Atherosclerosis of native arteries of extremities with rest pain, left leg
I70.229	Atherosclerosis of native arteries of extremities with rest pain, unspecified extremity
I70.261	Atherosclerosis of native arteries of extremities with gangrene, right leg
I70.262	Atherosclerosis of native arteries of extremities with gangrene, left leg
I70.269	Atherosclerosis of native arteries of extremities with gangrene, unspecified extremity
I70.263	Atherosclerosis of native arteries of extremities with gangrene, bilateral legs

Code	Description
I70.268	Atherosclerosis of native arteries of extremities with gangrene, other extremity
I73.9	Peripheral vascular disease, unspecified
M86.171	Other acute osteomyelitis, right ankle and foot
M86.172	Other acute osteomyelitis, left ankle and foot
M86.179	Other acute osteomyelitis, unspecified ankle and foot
M86.671	Other chronic osteomyelitis, right ankle and foot
M86.672	Other chronic osteomyelitis, left ankle and foot
M86.679	Other chronic osteomyelitis, unspecified ankle and foot
A48.0	Gas gangrene
E11.621	Type 2 diabetes mellitus with foot ulcer
L97.511	Non-pressure chronic ulcer of other part of right foot limited to breakdown of skin
L97.512	Non-pressure chronic ulcer of other part of right foot with fat layer exposed
L97.513	Non-pressure chronic ulcer of other part of right foot with necrosis of muscle
L97.518	Non-pressure chronic ulcer of other part of right foot with other specified severity
L97.514	Non-pressure chronic ulcer of other part of right foot with necrosis of bone
L97.519	Non-pressure chronic ulcer of other part of right foot with unspecified severity
L97.516	Non-pressure chronic ulcer of other part of right foot with bone involvement without evidence of necrosis
L97.515	Non-pressure chronic ulcer of other part of right foot with muscle involvement without evidence of necrosis
L97.529	Non-pressure chronic ulcer of other part of left foot with unspecified severity
L97.522	Non-pressure chronic ulcer of other part of left foot with fat layer exposed
L97.521	Non-pressure chronic ulcer of other part of left foot limited to breakdown of skin
L97.524	Non-pressure chronic ulcer of other part of left foot with necrosis of bone
L97.528	Non-pressure chronic ulcer of other part of left foot with other specified severity
L97.523	Non-pressure chronic ulcer of other part of left foot with necrosis of muscle
L97.526	Non-pressure chronic ulcer of other part of left foot with bone involvement without evidence of necrosis
L97.525	Non-pressure chronic ulcer of other part of left foot with muscle involvement without evidence of necrosis
E10	Type 1 diabetes mellitus
E11	Type 2 diabetes mellitus
E13	Other specified diabetes mellitus
E66	Overweight and obesity
I25.1	Atherosclerotic heart disease of native coronary artery

Code	Description
I25.9	Chronic ischemic heart disease, unspecified
I50.9	Heart failure, unspecified
I50.3	Diastolic (congestive) heart failure
I50.2	Systolic (congestive) heart failure
I50.4	Combined systolic (congestive) and diastolic (congestive) heart failure
I50.8	Other heart failure
I50.1	Left ventricular failure, unspecified
I10	Essential (primary) hypertension
I12	Hypertensive chronic kidney disease
I13	Hypertensive heart and chronic kidney disease
I11	Hypertensive heart disease
F11.1	Opioid abuse
F11.2	Opioid dependence
F17.2	Nicotine dependence
F33	Major depressive disorder, recurrent
F32	Depressive episode
F41	Other anxiety disorders
F43.1	Post-traumatic stress disorder (PTSD)
N18.6	End stage renal disease
N18.1	Chronic kidney disease, stage 1
N18.2	Chronic kidney disease, stage 2 (mild)
N18.5	Chronic kidney disease, stage 5
N18.4	Chronic kidney disease, stage 4 (severe)
N18.3	Chronic kidney disease, stage 3 (moderate)
Z87.891	Personal history of nicotine dependence
Z99.2	Dependence on renal dialysis
G62.9	Polyneuropathy, unspecified
G62.0	Drug-induced polyneuropathy
G57.00	Lesion of sciatic nerve, unspecified lower limb
G58.9	Mononeuropathy, unspecified
Z89.43	Acquired absence of foot
Z89.51	Acquired absence of leg below knee
Z89.61	Acquired absence of leg above knee
F41.1	Generalized anxiety disorder
**Procedural variable (CPT)**	
64905	Nerve pedicle transfer; first stage

Note. ICD-10-CM = International Classification of Diseases, Tenth Revision, Clinical Modification; CPT = Current Procedural Terminology.

Supplemental Table S2. ICD and CPT codes used for outcome analyses.

Outcome	Code(s)
Residual limb pain	G89.28, M79.60, M79.67, M79.672, M79.605, M79.66, M79.662, M79.606, M79.65, M79.651, M79.652, M79.659, M79.669, M79.661, M79.671, M79.674, M79.675, M79.673, M79.676, T87.33, T87.34
Phantom limb pain	G54.6, G54.7
Non-narcotic analgesic use	72625, 704, 7531, 39786, 25480, 187832, 4493, 32937, 2556, 321988
Stump/amputation revision, complications, or infection	27594, 27881, 27882, 27884, 27880, 27888, 27596, T87.43, T87.44, T87.53, T87.54, T87.89, T81.4
Opioid use	CN101
Death	Deceased (TriNetX platform-level mortality indicator)
Reduced ambulatory status	Z99.3, Z74.01, Z74.009

Note. Neurogenic stump pain, phantom limb pain, and non-ambulatory status were defined using ICD-10-CM codes; non-narcotic analgesic use using RxNorm codes; opioid use using TriNetX generic drug class CN101; stump/amputation revision, complications, or infection using CPT and ICD-10-CM codes; and death using the TriNetX platform-level mortality indicator. CPT = Current Procedural Terminology; ICD-10-CM = International Classification of Diseases, Tenth Revision, Clinical Modification; RxNorm = normalized naming system for medications.

Supplemental Table S3. Kaplan–Meier statistics for the three remaining outcomes that were not plotted in prior figures.

Time Interval	Outcome 3: Opioids			Outcome 4: Non-narcotics			Outcome 6: Reduced Ambulation		
	Hazard ratio	CI (95%)	p value	Hazard ratio	CI (95%)	p value	Hazard ratio	CI (95%)	p value
0-3 months	0.89	(0.86, 0.93)	**< 0.0001**	1.01	(0.97, 1.05)	0.61	1.02	(0.95, 1.10)	0.54
3-6 months	0.92	(0.87, 0.98)	**0.002**	1.04	(0.97, 1.11)	0.20	1.10	(0.98, 1.24)	0.10
6-12 months	0.88	(0.82, 0.94)	**< 0.0001**	1.00	(0.93, 1.08)	0.88	1.12	(0.99, 1.27)	0.07
1+ years	0.88	(0.84, 0.93)	**< 0.0001**	0.95	(0.90, 1.01)	0.07	1.10	(1.00, 1.20)	0.06
2+ years	0.87	(0.82, 0.93)	**0.001**	0.90	(0.84, 0.96)	< 0.0001	1.00	(0.89, 1.12)	0.94
5+ years	0.82	(0.74, 0.91)	**< 0.0001**	0.90	(0.80, 1.01)	**0.047**	0.99	(0.83, 1.20)	0.95

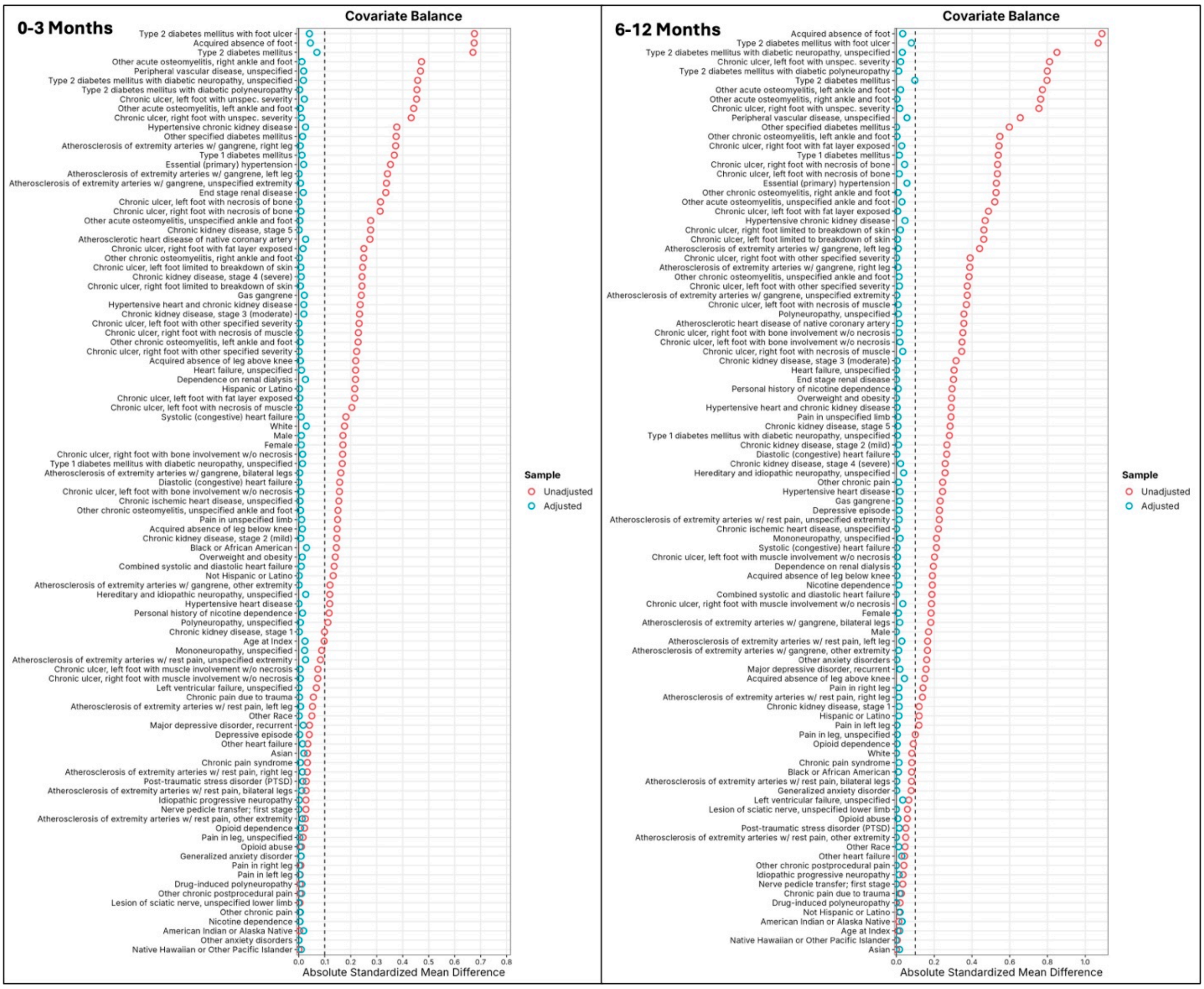
0-3 Months
Covariate Balance
Absolute Standardized Mean Difference
Sample
Unadjusted
Adjusted
6-12 Months
Covariate Balance
Absolute Standardized Mean Difference
Sample
Unadjusted
Adjusted

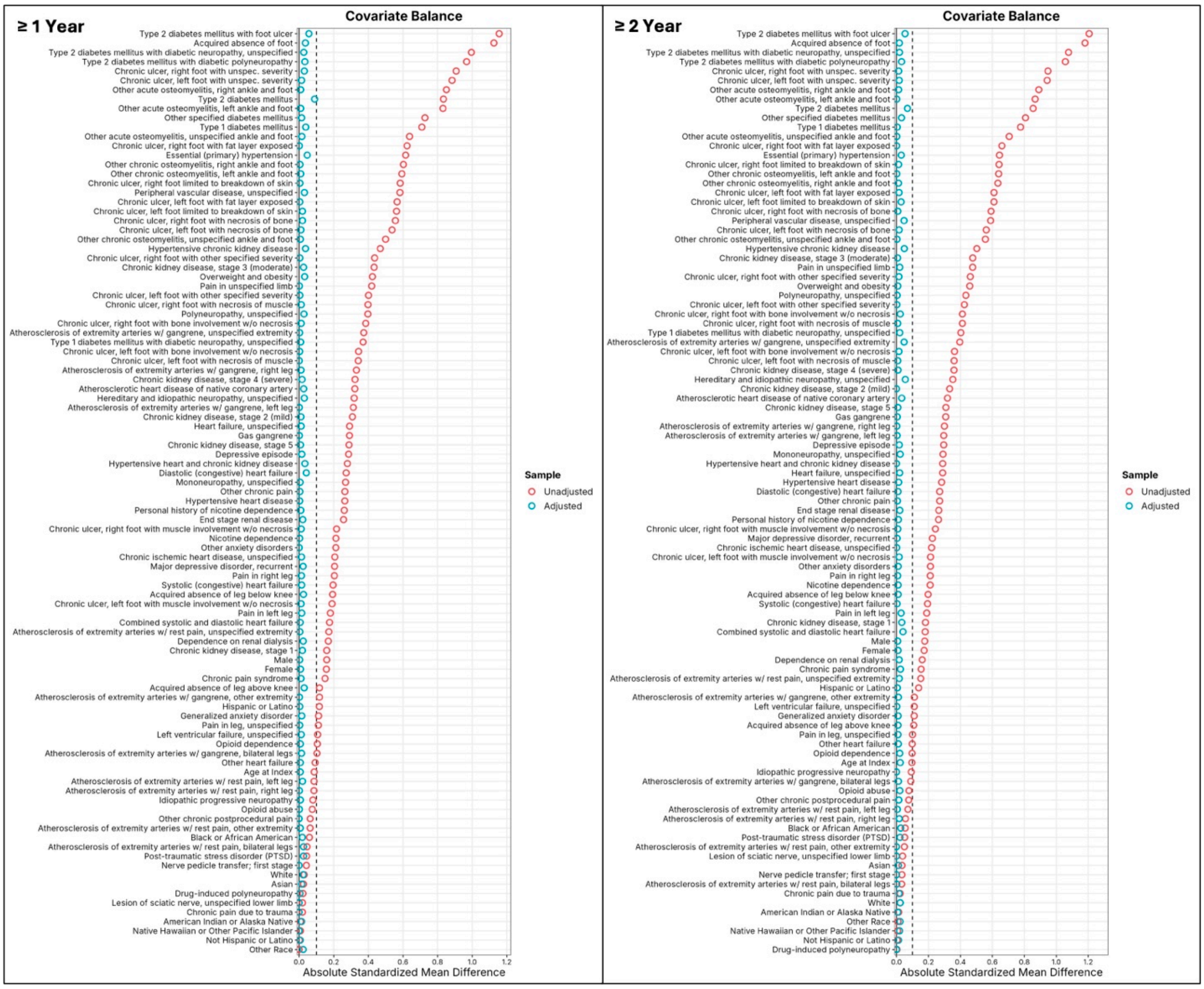

Supplemental Figure S1. The remaining SMD tables are presented. The set caliper at 0.1 is indicated by vertical dotted line.
